# Understanding and Managing Pineal Parenchymal Tumors of Intermediate Differentiation: An In-Depth Exploration from Pathology to Adjuvant Therapies

**DOI:** 10.3390/jcm13051266

**Published:** 2024-02-23

**Authors:** Andrea Bianconi, Flavio Panico, Bruna Lo Zito, Andrea Do Trinh, Paola Cassoni, Umberto Ricardi, Diego Garbossa, Fabio Cofano, Cristina Mantovani, Luca Bertero

**Affiliations:** 1 Neurosurgery Unit, Department of Neuroscience, University of Turin, 10126 Turin, Italy; 2 Radiation Oncology Unit, Department of Oncology, University of Turin, 10126 Turin, Italy; 3 Pathology Unit, Department of Medical Sciences, University of Turin, 10126 Turin, Italy

**Keywords:** pineal region, pineal parenchymal tumor, pineal gland, biopsy, intensity modulation radiation therapy, stereotactic radiosurgery, craniospinal irradiation

## Abstract

Background: Pineal parenchymal cell tumors constitute a rare group of primary central nervous system neoplasms (less than 1%). Their classification, especially the intermediate subtype (PPTIDs), remains challenging. Methods: A literature review was conducted, navigating through anatomo-pathological, radiotherapy, and neurosurgical dimensions, aiming for a holistic understanding of these tumors. Results: PPTIDs, occupying an intermediate spectrum of malignancy, reveal diverse histological patterns, mitotic activity, and distinct methylation profiles. Surgical treatment is the gold standard, but when limited to partial removal, radiotherapy becomes crucial. While surgical approaches are standardized, due to the low prevalence of the pathology and absence of randomized prospective studies, there are no shared guidelines about radiation treatment modalities. Conclusion: Surgical removal remains pivotal, demanding a personalized approach based on the tumor extension. This review underscores the considerable variability in treatment approaches and reported survival rates within the existing literature, emphasizing the need for ongoing research to better define optimal therapeutic strategies and prognostic factors for PPTIDs, aiming for further and more detailed stratification among them.

## 1. Introduction

Pineal parenchymal cell tumors (PPT) are a rare group of tumors representing less than 1% of all primary central nervous system neoplasms. Originating from pineocytes or their precursor cells, these tumors pose unique challenges both during the diagnostic assessment and clinical management. The World Health Organization (WHO) classification stratifies PPTs into distinct entities, ranging from the well-differentiated pineocytomas to the highly malignant pineoblastomas [1]. Among them, the intermediate category of pineal parenchymal cell tumors of intermediate differentiation (PPTID) remains a critically debated subset, presenting a spectrum of histologic features that defy easy categorization [2].

Tackling the management of these tumors remains a complex endeavor, primarily due to their rarity and the resulting limited pool of comprehensive studies. The inherent clinical heterogeneity exhibited by PPTs adds an additional layer of complexity. In this review, we delve into the intricacies of PPTs, emphasizing the histologic and immunohistochemical nuances that underpin their classification, and consequently their treatment. From the initial characterization by Schild et al. in 1993 to their formal inclusion in the WHO classification in 2000, PPTIDs have emerged as a distinct subgroup, encompassing both low and high-grade variants [3,4].

This review places a particular emphasis on the importance of a multidisciplinary approach, exploring anatomo-pathological, radiotherapeutic, and neurosurgical aspects. Through this lens, we aim to provide a comprehensive understanding of the clinical landscape, shedding light on the challenges in diagnosis and management while paving the way for future research endeavors.

## 2. Pathological Features of Pineal Parenchymal Tumors

According to the latest 2021 WHO classification of central nervous systems, two entities are defined at the opposite ends of the spectrum of pineal parenchyma tumors: pineocytoma (PC), a well-differentiated neoplasm, and pineoblastoma (PB), a poorly differentiated, aggressive neoplasm [1]. The pineal tumor of intermediate differentiation (PPTID) is located in the middle, representing a less defined group of neoplasms [5,6].

### 2.1. Pineocytomas

Pineocytoma was defined by the WHO in 2021 as a Grade 1 entity—a well-differentiated pineal parenchymal neoplasm exhibiting expansile growth that can result in compression of adjacent structures, leading to variable signs and symptoms [7]. The cut surface shows a well-circumscribed homogeneous or granular mass with a greyish-tan appearance. Histologically, it presents as a moderately cellular neoplasm composed of small, round, blue, and mature cells organized in sheets or showing large pineocytomatous rosettes, a hallmark feature, not present in the normal pineal gland. Gangliocytic differentiation can be variably present and a pleomorphic variant has also been described [8].

Mitotic figures are rarely present in pineocytomas [9,10,11]. The mean Ki67, in most cases, is <1% [11,12,13]. Pineocytomas exhibit strong positivity for synaptophysin, neuron-specific enolase, and NFP [2,9,13,14,15,16]. Other markers have shown variable positivity, including class III beta-tubulin, microtubule-associated protein tau, and chromogranin-A [2,9,14,15]. On average, the interval between the onset of symptoms and surgery was four years for pineocytomas [5]. To date, there have been no reported cases of metastasis in patients affected by pineocytoma [8,17]. The five-year survival in this group ranges from 86% to 91% [8,17]. A review highlighted that the extent of surgical resection is the main independent prognostic factor [18]. Immunoexpression of CRX, a transcription factor, and ASMT, a fundamental enzyme in the synthesis of melatonin, serves as a sign of a biological link to pinealocytes [19,20,21]. There are no recurrent genetic mutations in pineocytomas [22,23], but they exhibit a distinct methylation profile [24].

### 2.2. Pineal Parenchymal Tumors of Intermediate Differentiation

Pineal tumors of intermediate differentiation are characterized by intermediate malignancy between pineocytoma and pineoblastoma [4,7]. Histologically, they are composed of diffuse sheets or large lobules of monomorphic round cells that appear more differentiated than those observed in pineoblastomas. They can show two main microscopic patterns: they can be densely lobulated with an endocrine-arranged vascularity or diffuse, mimicking oligodendroglioma or neurocytoma. The nuclei are round with moderate atypia and “salt and pepper” chromatin [3,8]. According to the WHO in 2021, Grade 2 or 3 can be assigned based on histopathological features, highlighting the intrinsic heterogeneity of this neoplasm [1].

PPTIDs are positive for synaptophysin [9,13,25], while showing variable positivity for NFP and chromogranin-A [2,9,16,26]. As in pineocytoma, CRX is expressed as well as ASMT/HIOMT, which acts as both a diagnostic and prognostic marker [19,20,21]. Mitotic activity ranges from low to moderate [7]. The mean proliferation index Ki67 is significantly different from pineocytomas and pineoblastomas, with values ranging from 3.5% to 16.1% [22,25,27,28]. PPTIDs are less aggressive neoplasms compared to pineoblastoma, with a higher probability of localized disease at diagnosis. A more favorable prognostic difference between these entities can be observed by comparing the median overall survival of PPTID against PB (165 months vs. 77 months) and progression-free survival (93 months vs. 46 months) [29]. Jouvet et al. and Fauchon et al. have proposed a prognosis-oriented classification of PPTIDs with mitotic count and neuronal differentiation assessed by anti-NFP immunohistochemistry [9,17]. Low-grade PPTID, corresponding to WHO grade 2, was defined as having <6 mitosis per 10 HPF and expression of NFP in many cells [9]. Five-year survival in this group was 74%, and relapse occurred in 26%, mostly in the first site of the neoplasm after some delay [17]. High-grade PPTID, corresponding to WHO grade 3, was defined as having <6 mitosis without NFP expression by immunohistochemistry or >6 mitosis with NFP expression. Five-year survival in this group was 39%, and relapse occurred in 53%, mostly outside the pineal region [9,17]. Low-grade and high-grade prognostic groups showed a difference in the Ki67 proliferation index (5.2% vs. 11.2%) [10]. Nevertheless, the latest WHO classification of CNS tumors acknowledges that definite histological grading criteria are still missing.

It has been demonstrated that PPTIDs can harbor KBTBD4 small in-frame insertions [30]. The copy-number profile of PPTIDs is relatively flat, with some cases of broad gains or losses, particularly chromosome imbalances resembling those observed in pineoblastomas, though minor [22,24]. PPTIDs have a distinct methylation profile that can be further distinguished into two subtypes whose prognosis is still to be established: PPTID-A and PPTID-B [24].

### 2.3. Pinealoblastomas

Pineoblastoma is a malignant Grade 4 neoplasm—a poorly differentiated, highly cellular, malignant embryonal neoplasm arising in the pineal gland. Upon gross examination, they appear as partially defined invasive masses—soft and friable, pinkish-grey. Pineoblastomas appear as small round blue tumors composed of highly cellular sheets of small cells without a defined pattern. They show irregular, hyperchromatic nuclei with an occasional small nucleolus, high nuclear-to-cytoplasmic ratio, scant cytoplasm, and faint cell borders [3,7].

Pinealoblastomas exhibit positivity for synaptophysin and NSE [9]. Staining positivity for NFP and chromogranin A is significantly less frequent compared to pineocytomas [9,16,31]. There is no loss of SMARCB1/INI1 staining in pineoblastomas, a useful feature to distinguish them from atypical teratoid rhabdoid tumors [32]. Pineoblastoma is a neoplasm characterized by a high mean proliferation index, ranging from 16.9% to 50.1% [10,13,21,22]. It stands out as the most aggressive neoplasm of the pineal region, with frequent craniospinal dissemination and extracranial metastasis [3,17,33,34]. In older series, overall survival in pineoblastoma was reported to be as low as 1.3 years; however, recent studies indicate a better median overall survival time, reaching 4.1–8.7 years [35,36]. Negative prognostic predictors for pineoblastoma include disseminated disease at diagnosis, young age, and partial surgical resection [37]. The prognosis of pineoblastoma is extremely unfavorable, with patients often succumbing within two years from diagnosis [5].

From a cytogenetic perspective, structural alterations of chromosome 1 have been observed, and there may be losses of chromosomes [2,6,7,14,17] with some rare focal gains [22,38,39]. Reports also mention copy number variations and/or mutually exclusive mutations of DICER1, DROSHA, and DGCR8 [24,40,41,42,43]. DNA methylation profiling has identified four subgroups of pineoblastomas: miRNA processing altered type 1, miRNA processing altered type 2, RB1 altered, and MYC/FOXR activated [24,41,43]. These subgroups carry prognostic implications, with the miRNA processing altered type 2 subtype showing an overall good prognosis, while the outcomes of RB1-altered and the MYC/FOXR2-activated subgroups are notably poor.

## 3. Clinical Insights and Radiological Aspects

PPTID clinical presentation is not different from other PPTs and the main symptoms are linked to the increase in the intracranial pressure caused by obstructive hydrocephalus [44]. Developing hydrocephalus is a direct consequence of the extension of the tumor in the posterior part of the third ventricle and the obstruction of the cerebrospinal fluid flow through the acqueduct of Sylvius. Less common are symptoms from compression of the superior colliculus, with eye movement disorders such as Parinaud syndrome [3].

Also regarding radiological aspects, PPTIDs serve as a bridge entity between pineocytomas and pineoblastomas, exhibiting intermediate characteristics between the two. Pineocytomas commonly appear as well-defined, homogeneous masses measuring less than 3 cm on CT, exhibiting hypo- to isointense signal intensity on T1-weighted MRI sequences, and matching the intensity of brain parenchyma on T2-weighted sequences, occasionally with cystic or calcified areas [45,46]. In contrast, pineoblastomas are often larger and irregular, invading adjacent brain tissue, leading to hydrocephalus. On CT, they appear slightly hyperdense with post-contrast enhancement and possible calcifications. MRI findings for pineoblastomas include isointensity to hypointensity on T1-weighted images, isointensity on T2-weighted images with areas of cyst formation or necrosis, vivid heterogeneous enhancement on post-contrast T1 images, and restricted diffusion on DWI (diffusion weighted imaging)/ADC (Apparent Diffusion Coefficient) with ADC values around 400–800 mm^2^/s [47]. In MR spectroscopy, an increase in choline and a decrease in N-acetylaspartate can be observed, with the possibility of detecting myoinositol. However, limited data exist regarding cerebral blood flow and cerebral blood volume, which may be increased in pineoblastomas [48].

The PPTIDs, being able to exhibit characteristic features of both the aforementioned tumors, typically present as well-defined, isodense to hyperdense masses on CT scans, often with observable calcifications, which, like all pineal parenchymal tumors, tend to be present and dispersed peripherally. On T1-weighted MRI, they appear isointense to slightly hyperintense, while T2-weighted images may show hyperintensity. Contrast-enhanced MRI may reveal heterogeneous enhancement [7]. PPTIDs may demonstrate local invasion and can obstruct cerebrospinal fluid flow, leading to obstructive hydrocephalus [49,50]. Heterogeneous signal intensity, reflecting variations in cellularity and tissue composition, and different patterns, such as lobulated or diffuse, may be observed [51].

## 4. Role of Neurosurgery

### 4.1. Management of Hydrocephalus

In the case of these tumors, obstructive hydrocephalus, a common issue with pineal region tumors, remains a primary concern at diagnosis. Addressing hydrocephalus promptly is essential. Treatment options include the use of a ventricular internal shunt or, preferably, an endoscopic third ventriculostomy (ETV) [52,53]. ETV is preferred because, in addition to relieving hydrocephalus, it offers the opportunity to perform a biopsy if the tumor protrudes into the posterior part of the third ventricle [54]. ETV is a safe procedure with a very low risk of complications, mostly related to the challenging control of potential bleeding in highly vascularized lesions [55].

### 4.2. Biopsy

Before engaging in multidisciplinary therapeutic discussions, obtaining tissue samples is of paramount importance. In many patients with hydrocephalus, a biopsy can be performed during the third ventriculostomy itself, particularly in cases of large tumors extending forward within the third ventricle cavity [53,56,57]. For other patients, a stereotactic biopsy is typically conducted under neuronavigation guidance [58]. However, performing biopsies in PRTs carries the risk of obtaining non-representative samples, especially in cases of mixed tumors containing different tumoral components [55,59]. Despite the complex venous anatomy in the vicinity (including the Galen vein and tributaries), the morbidity and mortality associated with PRT biopsies are comparable to those of other brain locations [60].

### 4.3. Surgical Excision

The primary approach for PPTIDs continues to be extensive microsurgical removal, considered the benchmark. This approach should always be discussed in a multidisciplinary setting, involving a neuro-oncologist, a radiation specialist, and a neurosurgeon. The choice of a specific surgical approach depends on the tumor’s extensions in relation to the Galen venous complex and the surgeon’s experience [61].

The most frequently utilized approaches during the past two decades have been the occipital transtentorial (OTT) and infratentorial supracerebellar (ITSC) approaches [62]. The suboccipital transtentorial approach is preferable for tumors extending upward and pushing the venous complex downward. Patients are typically positioned either sitting or in a three-quarter prone position (Park Bench). This approach provides direct access to the pineal region below the Galen venous complex. However, it requires delicate handling of bridging veins and carries a risk of visual field dysfunction and other complications [63].

The infratentorial supracerebellar approach offers a direct route for tumors extending posteriorly. It is often performed with the patient in a sitting position. This approach involves sacrificing one or two bridging veins between the superior surface of the cerebellum and the tentorium; this usually does not entail risks as these are expendable veins that do not drain the brainstem, although there is a minimal risk of cerebellar hemorrhage [64].

Various other surgical approaches are possible depending on the tumor’s lateral or anterior extension within the third ventricle, each with its associated risks and benefits. However, these approaches should be carefully considered based on each patient’s unique case. 

## 5. Radiotherapy

Radiotherapy represents a cornerstone treatment in the multidisciplinary management of pineal parenchymal tumors. However, the rarity of the disease makes it difficult to define a standard treatment. Most of the evidence, especially in the adult population, derives from retrospective studies or small case series (Table 1).

The spectrum of radiation therapy recommendations is quite broad, ranging from focal treatment to craniospinal irradiation, based on histology. Modern radiation techniques (radiosurgery or stereotactic radiotherapy, VMAT) offer the opportunity to tailor radiation dose to the tumor volume, sparing normal brain tissue with a deeper gradient dose between the target and surrounding organs at risk. Similarly, the wider spread of proton therapy might reduce radiation-induced toxicity, especially in craniospinal irradiation.

Historically, in well-differentiated pineocytomas, radiation therapy was used as focal treatment in recurrent disease. Recent studies using SRS as part of multimodal treatment or as salvage therapy with the administration of marginal doses ranging from 14 to 16 Gy show high local tumor control ranging from 80% to 100%, with PFS of 80–100% at 5 years (Table 2).

Mori et al. [78] reported in six pineocytoma patients treated with SRS a PFS of 80% at 5 years; Lekovic et al. [79] achieved 100% local tumor control in eight patients with a mean follow up ranging from 2 to 56 months. In the series by Wilson et al. [80], five patients with subtotally resected pineocytoma underwent SRS as adjuvant therapy in three cases and as curative treatment in two cases with local tumor control at 65 months, without any toxicity. A multicentric retrospective large series on pineal tumors reported a local control rate of 80% at 20 years for 26 pineocytomas [71].

On the other hand, in aggressive pineoblastomas, due to the high risk of cerebrospinal dissemination, craniospinal irradiation represents the standard adjuvant treatment in combination with chemotherapy, with a total dose of 24–36 Gy to the entire craniospinal axis and a tumor boost to 54–55.8 Gy in 1.8–2 Gy fractions.

Recently, a cohort analysis on 201 adult patients with pineoblastoma from the SEER database (1975–2016) was published [81], showing that radiation treatment improves 5-year OS regardless of surgical treatment (5-year OS of 77.3% in the radiotherapy group versus 63.2% in the no-radiotherapy group). In this context, adjuvant radiotherapy improves local tumor control and overall survival.

The role of radiation therapy remains unclear in the management of the subgroup of pineal tumors of intermediate differentiation (PPTID), due to the lack of evidence and heterogeneous biological behavior in grade 2–3 tumors. Table 1 summarizes the most relevant clinical series on PPTID patients.

Some reports tried to collect individual patient data from the literature to show clinical characteristics, patterns of care, survival outcomes connected to treatment strategy, and finally to find out prognostic factors to guide clinicians in clinical practice.

Mallick et al. in 2016 [51] published an individual patient data analysis, based on 29 retrospective studies involving 127 patients. Information regarding radiation treatment was available for 65 cases; adjuvant radiation therapy was employed in 46 cases. Most of the patients received local irradiation (32.6% of cases), 14 patients received craniospinal irradiation, 2 patients received whole ventricular irradiation, and 1 patient received whole brain irradiation. Radiosurgery was employed in four patients. Twenty-four patients had recurrence, including nine local and fifteen leptomeningeal. The 3-year PFS was 63.4%, and the 5-year PFS was 52.2%. Median overall survival was 14 years, with 3- and 5-year OS values of 91% and 84.1%, respectively. In univariate analysis, female sex and adjuvant radiation were associated with better overall survival (*p* = 0.009), with a median OS of 252 months in irradiated patients versus 168 months in the untreated group.

In summary, the management of PPTID varies widely in the literature, including heterogeneous radiation treatment modalities concerning volume and doses, depending on local practices and physician preferences. Radiotherapy is commonly recommended for subtotally removed PPTID or as adjuvant therapy in grade 3 tumors.

Concerning the optimal treatment volume, a prevalent approach involves focal irradiation covering the surgical bed, residual disease, and all areas of suspected infiltration, utilizing modern high-gradient techniques. The total dose typically ranges from 54 to 59.4 Gy in conventional fractionation. Whole ventricle irradiation has been explored to reduce the risk of spinal metastases while mitigating the adverse effects associated with craniospinal irradiation (CSI), considering PPTID’s malignancy level between pineocytoma and pineoblastoma.

Justin T. Low et al. [77] treated five adult patients with grade 3 PPTID using adjuvant radiotherapy after resection, incorporating whole ventricle irradiation up to 25.2 Gy in 1.8 Gy daily fractions delivered with IMRT. This was followed by a stereotactic boost to the resection bed of 25.2 Gy and a second boost to the residual tumor of 5.5–9 Gy, reaching a total dose of 55.8 Gy–59.4 Gy. Three of the five patients experienced favorable outcomes, while three had progressive disease, resulting in two deaths. These findings suggest the feasibility of reduced-dose ventricular irradiation for treating PPTIDs.

According to Tsubasa Watanabe et al. [69], whole ventricle irradiation (WVI) might also have a role in association with CSI in PPTIDs with spinal dissemination. Two of five patients in their retrospective review had cerebrospinal dissemination at diagnosis and underwent biopsy-only surgery followed by 36 Gy of CSI + 18 Gy of WVI. Although the median relapse-free and overall survival were 72.9 and 94.1 months, respectively (three complete responses, two partial responses and two recurrences after treatment), some patients experienced cerebral white matter abnormalities and cognitive disturbance due to the association with CSI.

## 6. Conclusions

Surgical removal, when feasible, remains the primary treatment for PPTIDs, providing the potential for a curative outcome. However, due to the complexity of these tumors and their anatomical location, these procedures necessitate skilled surgeons and meticulous preoperative planning to optimize outcomes. In cases where complete excision is not achievable, a biopsy approach, whether stereotactic or otherwise, becomes essential to consider a radiation treatment plan. Radiation therapy assumes a pivotal role, especially in higher-grade lesions. The evolution of modern techniques, such as stereotactic radiosurgery and proton therapy, offers tailored approaches to optimize efficacy while minimizing collateral damage. 

## Figures and Tables

**Table 1:** Studies involving PPTIDs and radiotherapy treatment. Type of radiotherapy treatment, the administered dose, and radiation-related toxicity are reported. Abbreviations: BT: brachitherapy; CSI: craniospinal irradiation; IMRT: intensity-modulated radiation therapy; SRS: stereotactic radiosurgery; WBI: whole brain irradiation; WVI: whole ventricular irradiation.

Article	RT	Technique	Dose	Radiotherapy Toxicity
Balossier et al. [65]	curative	SRS	SRS: 15.5 Gy (isodose 50%)	no
Kumar et al. [66]	adjuvant	1 CSI, 3 WBI	IMRT: 54 Gy, CSI: 36 Gy	no
Park et al. [67]	curative	2 SRS, 3 IMRT	SRS: 13.3 Gy (isodose 50%), IMRT 30 Gy/5 fr (isodose 80%)	not reported for PPTID
Hasegawa et al. [68]	salvage	1 SRS	mean marginal dose 14 Gy. maximum marginal doses 28 Gy	not reported for PPTID
Kunigelis et al. [44]	adjuvant, salvage	IMRT, SRS, CSI	not reported	not reported
Ito et al. [25]	adjuvant, salvage	4 IMRT, 1 CSI.	IMRT: 50 Gy/25 fr, CSI: 54.4/28 fr	1 decline in activities of daily living by radionecrosis
Watanabe et al. [69]	adjuvant, salvage	IMRT, CSI	IMRT: 54 Gy;CSI 36 Gy +18 Gy WVI	2 neurocognitive disorder, 2 hypopituitarism
Lu et al. [70]	adjuvant	IMRT	IMRT: 54 Gy	not reported
Iorio-Morin et al. [71]	curative, salvage	SRS	median marginal dose 17 Gy (isodose 50%), median maximum dose 34 Gy	focal neurological deficit 9%, parinaud syndrome 7%, hydrocephalus 3%
Raleigh et al. [72]	adjuvant, salvage	2 IMRT, 12 CSI	CSI: 36 Gy + 55.8 Gy boost on pineal gland or local RT on pineal region	Growth defects, endocrine dysfunction, infertility, cognitive deficits
Stoiber et al. [73]	adjuvant	IMRT	IMRT: 54 Gy	no
Lutterbach et al. [29]	adjuvant, curative	IMRT, SRS, I125BT	IMRT: 54 Gy.	not reported
Choque-Velasquez et al. [74]	adjuvant, salvage	1 I125BTafter biopsy, 1 SRS, 6 IMRT, 2 unknown	IMRT: 54 Gy; SRS: 14 Gy	mild neuropsychologic deficits, depression, double vision
Nam et al. [75]	adjuvant	12 CSI (5 proton, 7 IMRT), 3 local RT (1proton, 2 SRS).	not reported	not reported
Chatterjee et al. [76]	adjuvant, salvage	IMRT	IMRT: 50–54 Gy, CSI: 36 Gy	not reported
Low, J.T. et al. [77]	adjuvant	IMRT	IMRT: 55.8–59.4 Gy/1.8 Gy: WVI 25.2 Gy + bed SRS boost 25.2 Gy + residual SRS boost 5.4–9 Gy	5 fatigue, 1 nausea, 1 alopecia, 1 hyponatriemia
Fauchon et al. [17]	adjuvant, curative	12 CSI, 8 WBI, 18 IMRT, 6 SRS	CSI: 31 Gy + boost, WBI 32.4 Gy + boost, IMRT: 78.8 Gy Gr. 2 and 53 Gy Gr. 3	1 radionecrosis in the talamus after SRS, 1 encephalitis after WBI

**Table 2:** Disease progression, recurrence, and survival outcomes in PPT patients. Abbreviations: CR: complete response; LC: local control; LR: local recurrence; OS: overall survival; PD: progression disease; PR: partial response; SD: stable disease.

Article	Patient Number	Median Follow Up Time (Months)	Local Control and Recurrence	Progression Free Surivival	Overall Survival
Balossier et al. [65]	12 (6 PPTID Gr. 2)	24	100% SD	not reported	not reported for PPTID
Kumar et al. [66]	14 (4 PPTID)	21.5	50% CR, 50% PR	not reported for PPTID	100% OS rate at reported follow up
Park et al. [67]	9 (5 PPTID)	78.6	40% CR, 60% PR	not reported	100% OS rate at reported follow up
Hasegawa et al. [68]	16 (2 PPTID)	61	33.3% CR, 16.67% PR, 16.67% SD	not reported	100% OS rate at reported follow up
Kunigelis et al. [44]	9 PPTID: 5 Gr. 2, 4 Gr. 3	95.3	22.2%LC (60% Gr. 2—100% Gr. 3 recurrence)	50.5 months	100% OS rate at 5 years follow up
Ito et al. [25]	6 PPTID	41	66.7% CR, 16.7% PD	50% after mean 3 years	83.33% OS rate at reported follow up
Watanabe et al. [69]	5 PPTID	not reported	60% CR, 40% PR, 40% PD	72.9 months	median OS 94.1 months
Lu et al. [70]	103 PPTID: 63 Gr. 2, 40 Gr. 3	49–75	not reported	not reported	OS rate at 1–2–5 year: 70%–58%–54%,
Iorio-Morin et al. [71]	70 (7 PPTID)	47	50% LC	34 months	OS rate at 5 years follow up: 56%
Raleigh et al. [72]	75 (18 PPTID: 10 Gr. 2, 8 Gr. 3)	49	16.67% recurrence: 10% Gr. 2, 25% Gr. 3	82% and 65% after 5 and 10 years	OS rate at 5–10 years: 76% and 61%
Stoiber et al. [73]	14 (1PPTID)	84	100% LC	PPTID free from relapse after 84mo	100% OS rate at reported follow up
Lutterbach et al. [29]	101 (37 PPTID)	38	3–5–10 years LC 86%–79%–53%. 3–5–10 years Spinal control 93%–92%–81%	93 months	median OS 165 months
Choque-Velasquez et al. [74]	15 PPTID	39-248	66.7% CR, 20% PR	33.3% at last follow up	OS rate at 5–10 years: 92% and 71%
Nam et al. [75]	17 PPTID	62.6	43.75% recurrence	20.9 months	OS rate at 5 years follow up: 64.7%
Chatterjee et al. [76]	16 PPTID: 6 Gr. 2, 10 Gr. 3	12–127	Gr. 3: 20% LR, 10% Spinal recurrence. Gr. 2 LC 100%	3–127 months	81.25% OS at reported follow up (100% Gr. 2, 70% Gr. 3)
Low, J.T. et al. [77]	5 PPTID Gr. 3	min 36	60% PD	not reported	60% OS rate at reported follow up
Fauchon et al. [17]	76 (28 PPTID, 27 Gr. 2, 20 Gr. 3)	85	Gr. 2–Gr. 3: 26%–56% recurrence	51 months	OS rate at 5 years follow up: 74% Gr. 2, 39% Gr. 3

## Data Availability

No new data were generated in this study.
